# P-759. Re-examining Respiratory Isolation for Tuberculosis: A review of isolation practices for people diagnosed with tuberculosis at a major academic medical center in 2022-2023

**DOI:** 10.1093/ofid/ofae631.954

**Published:** 2025-01-29

**Authors:** Aliya Moreira, Dana Hassneiah, Ruvandhi Nathavitharana

**Affiliations:** Beth Israel Deaconess Medical Center, Brookline, Massachusetts; Beth Israel Deaconess Medical Center, Brookline, Massachusetts; Beth Israel Deaconess Medical Center, Brookline, Massachusetts

## Abstract

**Background:**

Respiratory isolation to reduce tuberculosis (TB) transmission is routinely implemented yet adversely affects employment, education, food/housing security, and mental health. The National TB Coalition of America recently published guidelines on shortened respiratory isolation for TB in community settings, recommending five days of isolation post initiation of effective treatment in most circumstances. We sought to examine current isolation practices for people diagnosed with TB at an academic medical center.

**Table 1:**
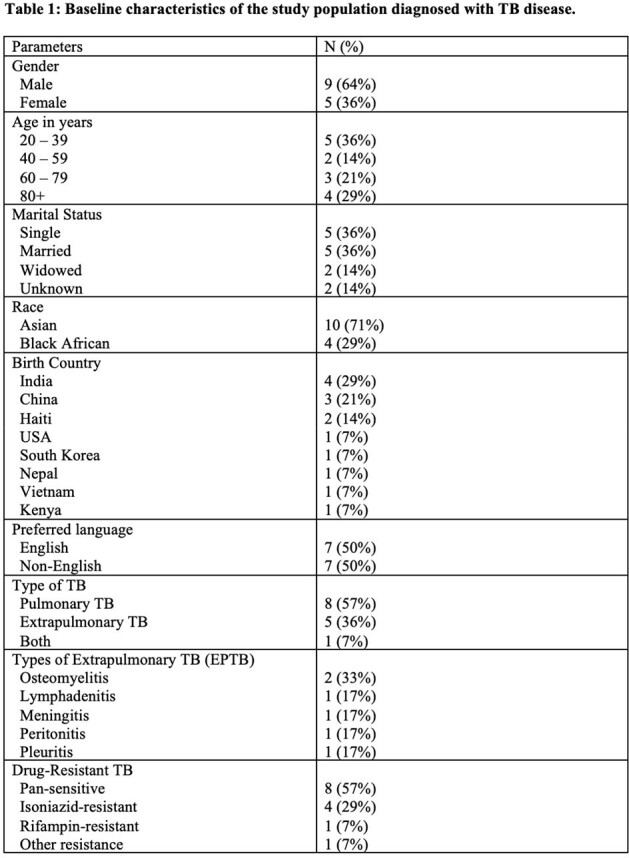
Baseline characteristics of the study group

**Methods:**

We undertook a retrospective record review to identify patients for whom the ICD-10 codes A15-A19 for TB had been utilized at Beth Israel Deaconess Medical Center in Boston from 2022-2023. We included people with TB disease and extracted data that included duration of isolation, results of smear, culture, and Xpert MTB/RIF testing, timing of smear conversion, documented discharge requirements, and patient perspectives on isolation requirements. Quantitative data were analyzed descriptively. Qualitative data were analyzed thematically.

**Table 2:**
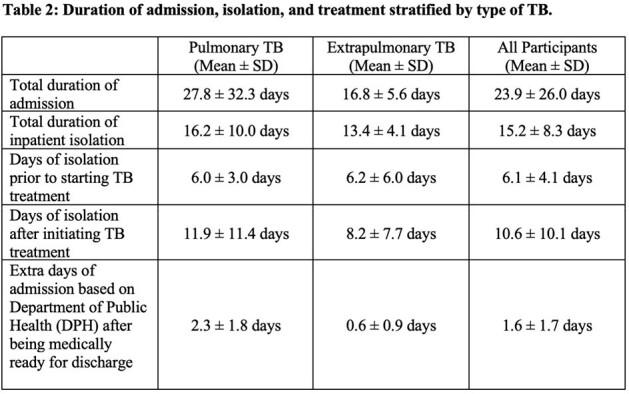
Duration of admission, isolation, and treatment in relation to different factors (Mean ± SD)

**Results:**

After reviewing 173 records, we identified 14 patients diagnosed with TB disease during a hospitalization, of whom eight had pulmonary TB (PTB), five had extrapulmonary TB (EPTB), and one had both PTB/EPTB. Six (43%) had some form of drug-resistance, predominantly isoniazid-resistant TB (29%). The majority (93%) were non-US born. The duration of inpatient isolation was 15.2 ± 8.3 days (mean ± standard deviation) (16.2 days for PTB, 13.4 days for EPTB). After TB treatment was initiated, patients remained in isolation for 10.6 ± 10.1 days (11.9 days for PTB, 8.2 days for EPTB). Inpatient stays were extended by 1.6 days after patients were documented as medically ready for discharge due to the need for Department of Public Health clearance. Qualitative data highlighted patients with TB experienced adverse events such as social isolation, stigma, language barriers, and delirium.

**Table 3:**
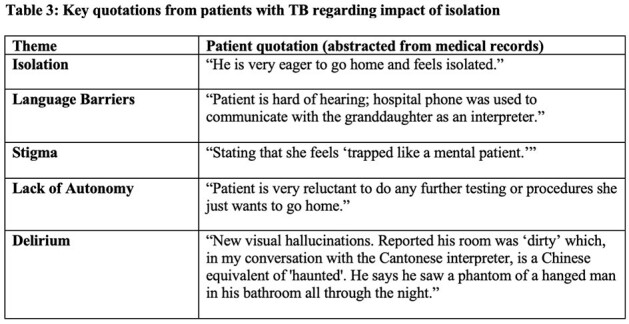
Key quotations from chart documentation regarding impact of isolation

**Conclusion:**

Our data highlight the long duration of inpatient isolation for people with TB, including after the initiation of treatment and in those with EPTB, and its negative impacts on patients. Implementation research is needed to understand the acceptability and reach of new isolation recommendations.

**Disclosures:**

**All Authors**: No reported disclosures

